# Qualitative Assessment of Longitudinal Changes in Phenocopy Frontotemporal Dementia

**DOI:** 10.3389/fneur.2019.01207

**Published:** 2019-11-14

**Authors:** Rozanna Meijboom, Rebecca M. E. Steketee, Lize C. Jiskoot, Esther E. Bron, Aad van der Lugt, John C. van Swieten, Marion Smits

**Affiliations:** ^1^Department of Radiology and Nuclear Medicine, Erasmus MC–University Medical Center Rotterdam, Rotterdam, Netherlands; ^2^Center for Clinical Brain Sciences, University of Edinburgh, Edinburgh, United Kingdom; ^3^Department of Neurology, Erasmus MC–University Medical Center Rotterdam, Rotterdam, Netherlands; ^4^Department of Radiology, Leiden University Medical Center, Leiden, Netherlands; ^5^Biomedical Imaging Group Rotterdam, Departments of Medical Informatics and Radiology, Erasmus MC–University Medical Center Rotterdam, Rotterdam, Netherlands

**Keywords:** frontotemporal dementia, diffusion tensor imaging, white matter, cognition, perfusion, gray matter, magnetic resonance imaging, cerebral blood flow

## Abstract

Phenocopy frontotemporal dementia (phFTD) shares core characteristics with behavioral variant frontotemporal dementia (bvFTD), yet without associated cognitive deficits and brain abnormalities on conventional magnetic resonance imaging (MRI), and without progression. Using advanced MRI techniques, we previously observed subtle structural and functional brain changes in phFTD similar to bvFTD. The aim of the current study was to follow these as well as cognition in phFTD over time, by means of a descriptive case series. Cognition, gray matter (GM) volume and white matter (WM) microstructure, and perfusion of 6 phFTD patients were qualitatively compared longitudinally (3-years follow-up), and cross-sectionally with baseline data from 9 bvFTD patients and 17 controls. For functional brain changes, arterial spin labeling (ASL) was performed to assess GM perfusion. For structural brain changes, diffusion tensor imaging was performed to assess WM microstructure and T1w imaging to assess GM volume. MRI acquisition was performed at 3T (General Electric, US). Clinical profiles of phFTD cases at follow-up are described. At follow-up phFTD patients showed clinical symptomatology similar to bvFTD, but had a relatively stable clinical profile. Longitudinal qualitative comparisons in phFTD showed some deterioration of language and memory function, a stable pattern of structural brain abnormalities and increased perfusion over time. Additionally, both baseline and follow-up cognitive scores and structural values in phFTD were generally in between those of controls and bvFTD. Although a descriptive case series does not allow for strong conclusions, these observations in a unique longitudinal phFTD patient cohort are suggestive of the notion that phFTD and bvFTD may belong to the same disease spectrum. They may also provide a basis for further longitudinal studies in phFTD, specifically exploring the structural vs. functional brain changes. Such studies are essential for improved insight, accurate diagnosis, and appropriate treatment of phFTD.

## Introduction

Phenocopy frontotemporal dementia (phFTD) is a clinical syndrome predominantly affecting men (1), that is much debated, as it shares core features with behavioral variant frontotemporal dementia (bvFTD) but does not follow its disease course. These core features are behavioral changes, such as apathy, behavioral disinhibition, and loss of insight (2). However, phFTD patients do not show the cognitive and brain abnormalities that are typical for bvFTD. The cognitive profile of phFTD patients may range from normal to suggesting bvFTD (3–6) but it appears stable over time, whereas in bvFTD rapid progression of cognitive deficits is evident (6–9). On conventional (structural) magnetic resonance imaging (MRI), phFTD patients show no or only mild abnormalities (7, 9) in the frontotemporal brain regions typically affected in bvFTD (10). Consequently, as a pathophysiological explanation is not yet available, diagnosis in phFTD is complicated. Patients often remain undiagnosed or may receive an uncertain psychiatric diagnosis, as PhFTD symptoms may be similar to those of psychiatric disorders, such as decompensated pre-existing personality disorders, late onset bipolar disorder and late-onset atypical psychosis (11–13). The issue of whether phFTD is a psychiatric or neurodegenerative disease could possibly be settled by observing brain abnormalities similar to bvFTD that progress over time, which have not been previously found in phFTD.

In our previous work (14, 15) we aimed to address this diagnostic concern and lack of neurological explanation by using advanced MRI techniques to investigate the possible presence of subtle brain abnormalities. Using diffusion tensor imaging (DTI) we observed white matter (WM) microstructure abnormalities in the frontal and temporoparietal lobes in phFTD similar to, but less pronounced than in bvFTD (14). Using an advanced post-processing method of structural imaging to explore gray matter (GM) volumes, we observed loss of GM volume in the right temporal lobe compared with healthy participants (15). Additionally, we observed a continuum of regional—especially frontotemporal—GM volumes ranging from normal in healthy participants to abnormal in bvFTD, with phFTD in between, with volumes not significantly different from neither normal nor bvFTD (15). Using resting state functional MRI (rs-fMRI) we observed increased functional connectivity in phFTD compared with healthy participants, in nearly all regions of the default mode network (DMN). This was similar to but more pronounced than in bvFTD (14). Using arterial spin labeling (ASL) we observed left frontal hyperperfusion in comparison with bvFTD and healthy participants (15). These findings suggest that subtle brain abnormalities are present in phFTD. Furthermore, psychiatric assessment ruled out a psychiatric diagnosis for this sample. As these abnormalities are similar to bvFTD—in addition to their similar symptomatology and lack of psychiatric diagnosis—we suggested that phFTD and bvFTD may belong to the same disease spectrum. To our knowledge, these subtle brain abnormalities as detected by advanced neuroimaging techniques have not been studied in phFTD elsewhere, and neither have they been longitudinally assessed. In order to gain more insight into possible longitudinal brain changes in phFTD and to acquire more support for the notion that phFTD and bvFTD may belong to the same disease spectrum, we present clinical reports and qualitatively describe findings from advanced MRI and neuropsychological examination of six phFTD patients at 3-years follow-up. We hypothesized that the more subtle brain abnormalities and cognitive impairment in phFTD at follow-up would show a slight deterioration in comparison with baseline, yet not fulfilling bvFTD criteria and still being in-between bvFTD and healthy participants.

## Methods

### Participants

All patients were recruited in the Alzheimer Center Rotterdam. We included phFTD patients (aged 40–75 years) with prominent behavioral changes interfering with social functioning, that were similar to bvFTD symptomatology (e.g., disinhibition, apathy, stereotypy); and without reported progression on neuropsychological and neurological assessment and MRI 1 year after initial routine diagnostic workup. From a larger study in our center, we included patients (aged 45–70 years) with a diagnosis of early-stage bvFTD (16)—based on neurological, neuropsychological and radiological assessment—and a Mini-Mental State Examination (17) (MMSE) score of ≥20. These patients meet the criteria for probable bvFTD. Patients with other neurological disorders, past or current substance abuse or other psychiatric diagnosis were excluded. Additionally, phFTD patients with a diagnosis of dementia or missing heteroanamnesis were also excluded.

Healthy controls (aged 60–70 years), without neurological or psychiatric history, were recruited through advertisement. They were matched for gender with phFTD patients and for age with all patients.

The study was approved by the local medical ethics committee. All participants gave written informed consent for participation in the study and publication of the case studies.

### Participant Assessment

PhFTD patients received an MRI scan, neuropsychological examination, MMSE, psychiatric assessment and genetic testing of the *C9ORF72* repeat expansion at baseline. Psychiatric assessment was performed to rule out major psychiatric disorders other than dementia that could explain the current behavioral changes present in phFTD, specifically focusing on late-onset psychotic disorders, manic episodes, major depression disorder, anxiety disorders and autism spectrum disorder. The assessment was conducted by a qualified psychiatrist and consisted of an interview with the patient and their caregiver, during which the Brief Psychiatric Rating Scale [BPRS (18); Dutch translation (19)] was administered. Genetic testing was performed because FTD with the *C9ORF72* repeat expansion may occasionally mimic phFTD, with cognitive deficits remaining stable over years (20). Such cases would have to be excluded from our phFTD sample.

For convenience we will refer to the above described group as “baseline phFTD.” At 3-years follow-up, phFTD patients received an MRI scan, neuropsychological examination and MMSE. BvFTD patients and controls underwent an MRI scan, neuropsychological examination and MMSE only once.

### Image Acquisition and Assessment

Scanning was performed on two 3T GE Discovery MR750 systems (GE Healthcare, Milwaukee, WI, US) with identical protocols. PhFTD patients (both at baseline and follow-up) and 9 controls (baseline) were scanned on one, and bvFTD patients and 8 controls (baseline) on the other scanner.

For clinical radiological assessment by a neuroradiologist and GM structural assessment, a high-resolution 3-dimensional (3D) inversion recovery (IR) fast spoiled gradient-echo (FSPGR) T1-weighted scan was acquired [duration 4.41 min; field of view (FOV) 240 mm; inversion time (TI) 450 ms; echo time (TE) 3.06 ms; repetition time (TR) 7,904 ms; acquisition matrix 240 × 240 mm^2^; slice thickness 1 mm; isotropic voxel size 1 mm^3^; 176 slices; array spatial sensitivity encoding technique acceleration (ASSET) factor 2, flip angle 12°].

For cerebral blood flow (CBF) measurements, ASL scans were acquired using whole-brain 3D pseudo-continuous ASL (p-CASL), which is currently the recommended sequence for clinical use (21) (duration 4.29 min; FOV 240 mm; TE 10.5 ms; TR 4,632 ms; isotropic voxel size 3.3 mm^3^; 36 axial slices; interleaved fast spin-echo stack-of-spiral readout 512 sampling points on eight spirals; background suppression; labeling duration 1,450 ms; number of excitations 3; post-labeling delay 1,525; labeling plane 9 cm below anterior commissure–posterior commissure line).

For WM microstructure diffusion measurements, DTI scans with full coverage of the supratentorial brain were acquired using a spin echo echo planar imaging (EPI) sequence (duration 3.50 min; FOV 240 mm; TE set to minimum with mean 84.04 ms (range: 81.9–90.8 ms; TE mean and range based on baseline scans); TR 7,925 ms; acquisition matrix 128 × 128 mm, slice thickness 2.5 mm; voxel size 1.88 × 1.88 × 2.5 mm^3^; 28 volumes with 59 axial slices each; 3 non-diffusion weighted volumes; 25 diffusion-weighted directions; maximum *b*-value 1,000 s/mm^2^; ASSET factor 2; flip angle 90°).

GM volumes and CBF were post-processed according to the methods previously described in Bron et al. (22) and applied in Steketee et al. (15) and Meijboom et al. (14). In brief, SPM8 was used for segmentation of GM, WM and CSF tissue maps, which were then transformed to ASL image space using Elastix registration software (23) for partial volume correction. Partial volume corrected ASL images were quantified using the single-compartment model (21). A multi-atlas approach was used to establish whole-brain and regional GM volumes expressed as percentage of intracranial volume (%ICV), and whole-brain and regional mean CBF (in ml/100 g gm/min). Appropriate regional CBF values were averaged to establish mean CBF for each brain lobe.

WM diffusion was post-processed using tractography according to the methods previously applied in Steketee et al. (24). In brief, automated probabilistic tractography [AutoPtx (25)] was performed to apply DTIFit (26) for fitting the tensor, and to apply BEDPOSTX and PROBTRACKX (26, 27) for creating WM tract images. Median fractional anisotropy (FA) was then established for bilateral WM tracts: anterior thalamic radiation, cingulate and hippocampal cingulum, inferior fronto-occipital fasciculus, inferior and superior longitudinal fasciculus, uncinate fasciculus, and forceps minor and major.

### Neuropsychological Data Acquisition and Assessment

Cognitive testing was performed and evaluated by a neuropsychologist at Alzheimer Center Rotterdam. These cognitive tests and associated cognitive domains used for qualitative assessment are listed in Table 1. The patients and controls described here were recruited as part of two different studies, with each study administering different additional cognitive tests. The scores for these additional tests are not reported (Table 1).

**Table 1:** Cognitive domains and their specific neuropsychological tests used to assess cognitive functioning in patients and controls.

**Cognitive domain**	**Neuropsychological test**	**Included in cognitive domains for qualitative comparison**
Attention and executive functions	Trail Making Test (TMT) (28)	✓
	Stroop color-word task (29)	✓
	Categorical and letter fluency test (30)	✓
	Modified Wisconsin Card Sorting test (WCST) (31)	✓
	Letter Digit Substitution Test (LDST) (32)	✓
Language	Boston Naming Test (60 items) (33)	✓
Memory	15 Words Test (34)	✓
	Digit Span of the Wechsler Adult Intelligence Scale third edition (WAIS-III) (35)	×
	Mini-Mental State Examination (MMSE) orientation questions (17)	✓
	Visual Association Test (36)	×
Visuoconstructive functioning	Clock drawing (37)	✓
Social cognition	Ekman Faces (38)	×

*The third column specifies which tests were available for all participants and thus included in the qualitative comparison of cognitive domains across participants*.

Test scores were transformed to z-scores using the mean and standard deviation of controls as a reference. For domains assessed with more than one test (Table 1), z-scores were averaged to establish one score per domain (SPSS21.0, New York, USA).

### Qualitative Analysis of Baseline vs. Follow-Up Measures

Seven phFTD patients were included for baseline. Upon follow-up, one phFTD patient was excluded due to lack of follow-up data: the family of the patient did not want him to participate. Six phFTD patients underwent follow-up assessment, one of whom did not undergo MRI due to a pacemaker placed after the baseline visit. Only neuropsychological examination was performed in this patient.

Data and results from reference groups (phFTD at baseline, cross-sectional bvFTD and control data) previously described (14, 15) were used for qualitative comparison with phFTD patients at follow-up. The phFTD patient that was excluded from follow-up was for that reason also excluded from the baseline group. Three bvFTD patients were excluded due to incomplete data: two had missing neuropsychological data and one had ASL scans of unusable quality.

In total, cognitive, DTI, ASL, and structural imaging data of 6 phFTD patients at baseline and follow-up (5 for MRI data), 9 bvFTD patients and 17 controls were used for qualitative comparisons (i.e., no statistical tests were used). For each group, we reported mean and range of cognitive domain z-scores, WM tract FA, whole-brain GM volume (%ICV) and whole-brain and regional CBF per group (SPSS21, New York, USA). To gain insight on a case level, we additionally reported baseline and follow-up measurements for these variables for each case. Cases with a 10% difference between both time points were highlighted to separate these from cases with only minimal differences.

### Demographics of Previously Described Reference Groups (14, 15)

Age did not differ between phFTD at baseline, bvFTD and controls [*F*(2) = 0,856 *p* > 0.05; Welch-ANOVA test]. The ANOVA test for MMSE score was significant [*F*(2) = 4.035, *p* = 0.028], and *post-hoc* between-group comparisons showed a lower MMSE score in phFTD than in controls (*p* = 0.041) (SPSS21.0, New York, USA). See Table 2 for an overview of the demographics.

**Table 2:** Demographic characteristics of baseline reference groups used.

**Group**	***N***	**Mean age**	**Mean MMSE**	**Education level**
PhFTD	6 (6 male)	63.8 (59-70)	26.3 (24-28)	4.5 (4-6)
BvFTD	9 (4 male)	60.1 (43-69)	27.1 (24-30)	4.6 (3-6)
Controls	17 (17 male)	64.1 (56-69)	28.3 (25-30)	5.5 (4-7)

*phFTD, phenocopy frontotemporal dementia; bvFTD, behavioral variant frontotemporal dementia; N, sample size. Values given as mean (range). MMSE, Mini-Mental State Examination. Education levels reported using the Dutch Verhage education scale (1964): with 1 = <6 years of primary education, 2 = completed primary education, 3 = primary education and <2 years of secondary education, 4 = completed low-level secondary education, 5 = completed middle-level secondary education, 6 = completed high-level secondary education, 7 = university education (39, 40)*.

None of the phFTD patients received an alternative psychiatric diagnosis at baseline that could explain their behavioral symptoms. Additionally, none carried the *C9ORF72* repeat expansion.

## Results

Follow-up neuropsychological assessment was performed with a mean of 2.93 years (range 2.67–3.08) after baseline neuropsychological assessment. For MRI at follow-up this was 2.98 (range 2.79–3.15).

### Case Descriptions

All cases showed a clinical profile suggestive of bvFTD. Increased behavioral changes were reported by the patient or partner in 5 out of 6 patients; for one patient this information was not available. Neuropsychological reports stated that some progression of cognitive abnormalities was present in five patients in comparison with baseline, but evident progression was lacking. One patient showed a normal cognitive profile similar to his baseline profile. Radiological reports showed no progression of brain abnormalities in 4 out of 5 patients, and varied from normal to global abnormalities unspecific for bvFTD. In one patient, a mild progression of global abnormalities was reported. A summary of MRI abnormalities can be found in Table 3.

**Table 3:** Summary of follow-up radiological assessment for each phFTD case.

	**Case 1**	**Case 2**	**Case 3**	Case 4^*^	**Case 5**	**Case 6**
Changes (T1–T0)	No	Yes	No	–	No	Yes
Gray matter atrophy	None	GCA 1 >>	GCA 1	GCA 1	None	Minimal frontal atrophy
White matter changes	None	Fazekas 3	None	Fazekas 1	None	None
Infarct	None	None	None	None	Cortical, lacunar	None
Microbleeds	None	None	None	None	None	3>>

* *Baseline scan, follow-up was unavailable due to pacemaker*.

*>> Indicates an increase in comparison with baseline*.

*T1, follow-up; T0, baseline; phFTD, phenocopy frontotemporal dementia; GCA, global cortical atrophy*.

#### Case 1

Patient 1 was a 64-years-old male in whom behavioral changes were first noticed 15 years previously. Major complaints involved loss of empathy, increased and uncontrolled anger, loss of initiative, compulsivity, irritability, increased talking and moving. At follow-up, increased anger and irritability, and decreased empathy were reported by the partner.

The neuropsychological report at follow-up stated disorders of language (naming), divided attention and social cognition. Additionally, mild abnormalities in the executive functions and working memory/memory were observed. In comparison with the neuropsychological report at baseline the patient showed a very mild progression of language, memory and divided attention abnormalities. Although the clinical and neuropsychological profile suggested bvFTD, the protracted disease course and lack of evident progression of cognitive dysfunctioning did not support the clinical diagnosis of probable bvFTD.

The radiological report at follow-up stated that no GM atrophy or WM abnormalities were observed on conventional MRI.

#### Case 2

Patient 2 was a 73-years-old male in whom behavioral changes were first noticed at least 12 years previously. Major complaints included increased dependency on his partner, loss of initiative, apathy, increase of food intake, and the inability to cope with changes. At follow-up, a general worsening of complaints was reported by the partner, with which the patient disagreed.

The neuropsychological report at follow-up stated disorders of naming, task-switching, visuo-associative memory and emotion recognition. In comparison with baseline neuropsychological examination there was a decrease in memory functioning and inhibition. The other cognitive functions were of similar level to baseline.

The radiological report of conventional imaging at follow-up stated a mild increase of global GM atrophy [global cortical atrophy scale 1 (GCA)], and no regional GM atrophy. Additionally, it stated the presence of previously described WM lesions (Fazekas 3), which were most likely of vascular origin.

#### Case 3

Patient 3 was a 74-years-old male in whom behavioral changes were first noticed at least 8 years previously. Major complaints included angry and aggressive behavior, impulsivity, decreased empathy, sexual disinhibition, speech disinhibition, compulsive information gathering, and no symptom insight. At follow-up, no change in complaints was observed.

The neuropsychological report at follow-up stated weak scores on verbal memory and emotion recognition. Other domains showed average or just-below average scores. In comparison with baseline neuropsychological examination, emotion recognition was more abnormal, but in contrast, scores for a complex executive task were higher. Scores on the other domains showed no changes.

The radiological report of conventional imaging at follow-up stated no regional, but very mild global atrophy (GCA 1). No changes were observed in comparison with baseline MRI.

#### Case 4

Patient 4 was a 62-years-old male in whom behavioral changes were first noticed at least 10 years previously. Major complaints included severe forgetfulness, letter switching during speech, incorrect word use, general disinhibition, and disinhibition of speech specifically. At follow-up, the patient reported a general worsening of complaints.

Neuropsychological report at follow-up stated weak performance of divided attention, and below-average language performance, processing speed and working memory. The other domains, among which memory and social cognition, demonstrated average scores. In comparison with baseline neuropsychological examination there was progression in one attention and one processing speed task, while other scores remained stable.

The patient did not receive a follow-up MRI due to pacemaker placement after baseline. The radiological report of conventional imaging at baseline stated mild global atrophy (GCA 1), which was somewhat more pronounced in the parietal lobe. There were some WM lesions (Fazekas 1). Importantly, as the patient reported memory problems, the report stated that no hippocampal atrophy was observed.

#### Case 5

Patient 5 was a 67-years-old male in whom behavioral changes were first noticed 10 years previously. Major complaints included loss of empathy, impulsive purchases, increased intake of food, isolated behavior, unhappiness, increased anger triggered by minor events, loss of initiative, reduced vocabulary and forgetfulness. At follow-up, an increase in anger and decrease in interest and empathy was reported by both patient and partner.

The neuropsychological report at follow-up stated average and above-average performance on cognitive domains, with the exception of weak performance on emotion recognition. The report concluded that no cognitive disorders were objectified, and that there were no changes in comparison with neuropsychological examination at baseline.

The radiological report of conventional imaging at follow-up stated no cortical atrophy or WM lesions, other than known lacunar infarcts in the brainstem and a cortical infarct in the right parietal lobe. This profile was not different from MRI at baseline.

#### Case 6

Patient 6 was a 63-years-old male in whom behavioral changes were first noticed 20 years previously. Major complaints included short-term memory loss, reduced personal hygiene, verbal aggressiveness, inappropriate—e.g., sexual–remarks, word-finding problems, and loss of control concerning eating -mainly sweets-, and smoking. Alcohol intake was controlled by patient's partner, as it would be excessive otherwise. At follow-up, increased passive behavior was reported by the partner.

Neuropsychological report at follow-up stated disorders of executive functioning, attention and social cognition. Weak memory performance was observed for encoding of new material, retaining and recall. Visuoconstruction and praxis were abnormal. There were no evident disorders of language and orientation. In comparison with baseline neuropsychological examination, minimal progression was observed for executive functioning, social cognition and processing speed. However, overall the cognitive profile was stable in comparison with neuropsychological examination performed in 2003, 10 years prior to baseline study examination.

The radiological report of conventional imaging at follow-up stated minimal frontal atrophy, which did not show evident progression in comparison with baseline MRI. Additionally, 3 microbleeds were observed, of which a hypertensive origin was suggested.

### Qualitative Comparison of Baseline and Follow-Up

**Figure 1:**
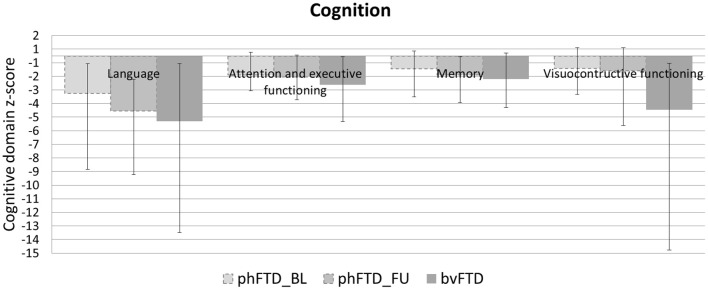
Mean cognitive domain z-scores for each group (baseline phFTD, follow-up phFTD, bvFTD). Z-scores are calculated using means of controls as a reference (i.e., control mean = 0, hence not visible in the graph). The range of scores is indicated by a minimum-maximum bar. Domains assessed were language, attention and executive functioning, memory, and visuoconstructive functioning. bvFTD, behavioral variant frontotemporal dementia; phFTD, phenocopy frontotemporal dementia; BL, baseline; FU, follow-up.

Patients with phFTD performed worse on follow-up neuropsychological assessment than at baseline for the domains language and memory. Follow-up and baseline scores for visuoconstructive functioning and attention and executive functioning were comparable. Follow-up phFTD scores for all domains (Table 4) were in between scores of bvFTD patients and controls (Figure 1). Any differences between individual results reported in Table 4 and clinical neuropsychological reports are due to different assessment strategies (i.e., calculation of domain scores vs. clinical (individual test) interpretation).

**Table 4:** Cognitive domain z-scores for phenocopy frontotemporal dementia (phFTD) cases at baseline and follow-up.

		**Case 1**	**Case 2**	**Case 3**	**Case 4**	**Case 5**	**Case 6**
Language	Baseline	−2.90	−8.33	−0.96	−2.12	−0.57	−1.73
	Follow-up	−5.62^*^	−8.72	−1.73^*^	−4.06^*^	−2.12^*^	−2.12^*^
Attention and executive functioning	Baseline	−1.80	−2.55	−0.92	−1.09	0.26	−2.28
	Follow-up	−1.64	−2.98^*^	−0.68^*^	−0.78^*^	0.07^*^	−3.22^*^
Memory	Baseline	−0.88	0.35	−1.12	−0.87	−0.14	−3.01
	Follow-up	−1.77^*^	−0.89^*^	−1.60^*^	−0.07^*^	−0.56^*^	−3.43^*^
Visuoconstructive functioning	Baseline	−0.54	−0.54	0.61	−0.54	−1.68	−2.83
	Follow-up	0.61^*^	−0.54	−0.54^*^	0.61^*^	−1.68	−5.11^*^

*More than 10% difference between baseline and follow-up is indicated by an asterisk*.

**Figure 2:**
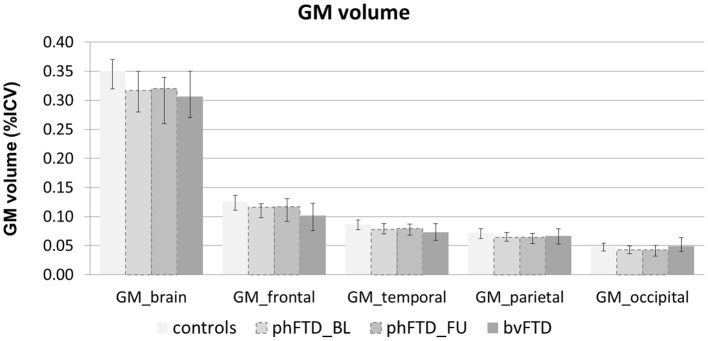
Whole-brain and regional gray matter (GM) volume expressed as percentage of intracranial volume (ICV) shown for each group (controls, baseline phFTD, follow-up phFTD, bvFTD). The range of scores is indicated by a minimum-maximum bar. bvFTD, behavioral variant frontotemporal dementia; phFTD, phenocopy frontotemporal dementia; BL, baseline; FU, follow-up.

**Figure 3:**
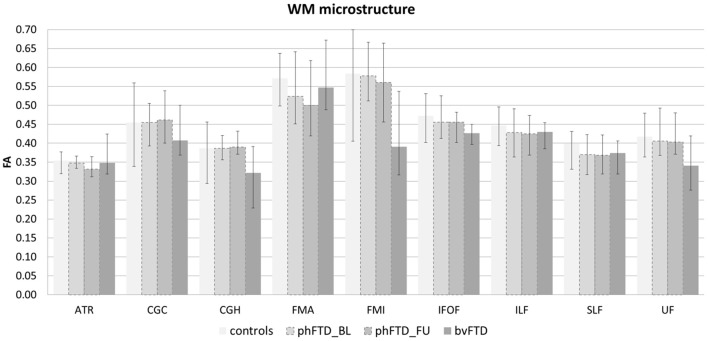
White matter (WM) microstructure represented by fractional anisotropy (FA) per WM tract shown for each group (controls, baseline phFTD, follow-up phFTD, bvFTD). The range of FA is indicated by a minimum-maximum bar. WM tracts included are the bilateral anterior thalamic radiation (ATR), bilateral cingulate cingulum (CGC), bilateral hippocampal cingulum (CGH), forceps major (FMA), forceps minor (FMI), bilateral inferior fronto-occipital fasciculus (IFOF), bilateral inferior longitudinal fasciculus (ILF), bilateral superior longitudinal fasciculus (SLF), and bilateral uncinate fasciculus (UF). bvFTD, behavioral variant frontotemporal dementia; phFTD, phenocopy frontotemporal dementia; BL, baseline; FU, follow-up.

**Figure 4:**
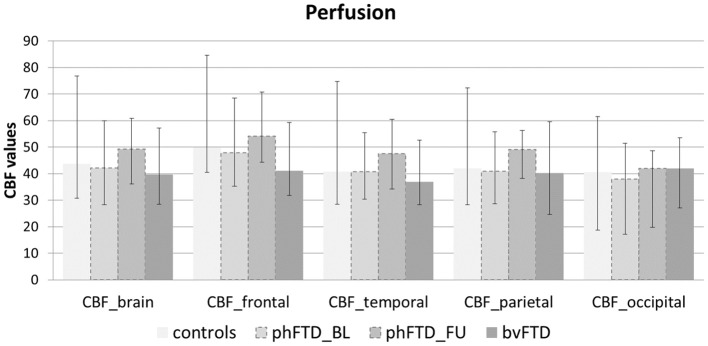
Whole-brain perfusion and lobar perfusion shown for each group (controls, baseline phFTD, follow-up phFTD, bvFTD). The range of perfusion is indicated by a minimum-maximum bar. CBF, cerebral blood flow; bvFTD, behavioral variant frontotemporal dementia; phFTD, phenocopy frontotemporal dementia; BL, baseline; FU, follow-up.

Whole-brain, frontal and temporal GM volume (Figure 2, Table 5) in phFTD upon follow-up were similar to baseline and in between volumes of bvFTD and controls. Parietal and occipital GM volume in phFTD at follow-up were also similar to baseline measures and lower than controls, similar to bvFTD for parietal GM, and lower than bvFTD for occipital GM. WM tract FA in follow-up phFTD was similar to baseline phFTD (Figure 3, Table 6), and generally in between bvFTD and controls. However, whole-brain and lobar perfusion in follow-up phFTD was increased in comparison with baseline phFTD (Figure 4, Table 5).

**Table 5:** Whole brain and regional gray matter (GM) volume (% intracranial volume) and cerebral blood flow (CBF in ml/100 g gm/min) for phenocopy frontotemporal dementia (phFTD) cases at baseline and follow-up.

		**Case 1**	**Case 2**	**Case 3**	**Case 5**	**Case 6**
Whole-brain GM volume (%ICV)	Baseline	0.3491	0.2759	0.3368	0.3165	0.3083
	Follow-up	0.3433	0.2589	0.3374	0.3215	0.3408^*^
Frontal lobe GM	Baseline	0.1220	0.0979	0.1212	0.1196	0.1193
	Follow-up	0.1190	0.0919	0.1205	0.1219	0.1309
Temporal lobes GM	Baseline	0.0875	0.0707	0.0792	0.0825	0.0706
	Follow-up	0.0867	0.0681	0.0793	0.0838	0.0784^*^
Parietal lobes GM	Baseline	0.0727	0.0575	0.0712	0.0573	0.0617
	Follow-up	0.0707	0.0538	0.0711	0.0583	0.0690^*^
Occipital lobes GM	Baseline	0.0466	0.0361	0.0493	0.0399	0.0410
	Follow-up	0.0462	0.0321^*^	0.0506	0.0404	0.0440
Whole-brain CBF	Baseline	37.49	59.88	28.36	42.60	42.19
	Follow-up	54.45^*^	60.86	36.18^*^	44.14	50.54^*^
Frontal lobes CBF	Baseline	39.49	68.45	35.28	49.22	46.96
	Follow-up	55.86^*^	70.78	47.81^*^	44.22^*^	52.26^*^
Temporal lobes CBF	Baseline	36.93	55.39	30.30	40.44	40.70
	Follow-up	57.48^*^	60.44	34.24^*^	42.13	43.52
Parietal lobes CBF	Baseline	37.82	55.83	28.68	39.30	42.54
	Follow-up	53.20^*^	56.27	38.17^*^	41.71	56.14^*^
Occipital lobes CBF	Baseline	35.18	51.49	17.11	47.56	38.22
	Follow-up	46.49^*^	47.71	19.75^*^	48.61	46.78^*^

*More than a 10% difference between baseline and follow-up is indicated by an asterisk*.

**Table 6:** White matter (WM) tract fractional anisotropy for phenocopy frontotemporal dementia (phFTD) cases at baseline and follow-up.

		**Case 1**	**Case 2**	**Case 3**	**Case 5**	**Case 6**
Anterior thalamic radiation L	Baseline	0.35	0.32	0.37	0.35	0.36
	Follow-up	0.34	0.30	0.36	0.34	0.30^*^
Anterior thalamic radiation R	Baseline	0.33	0.35	0.38	0.33	0.34
	Follow-up	0.32	0.32	0.37	0.34	0.33
Cingulate cingulum L	Baseline	0.51	0.45	0.48	0.41	0.50
	Follow-up	0.52	0.45	0.54^*^	0.42	0.52
Cingulate cingulum R	Baseline	0.44	0.38	0.51	0.38	0.50
	Follow-up	0.46	0.39	0.50	0.38	0.43^*^
Hippocampal cingulum L	Baseline	0.42	0.35	0.35	0.36	0.38
	Follow-up	0.43	0.31^*^	0.38	0.39	0.40
Hippocampal cingulum R	Baseline	0.43	0.36	0.40	0.40	0.42
	Follow-up	0.40	0.38	0.40	0.43	0.37^*^
Forceps major	Baseline	0.57	0.45	0.64	0.50	0.46
	Follow-up	0.52	0.44	0.64	0.50	0.49
Forceps minor	Baseline	0.67	0.51	0.64	0.55	0.51
	Follow-up	0.66	0.48	0.68	0.56	0.52
Inferior fronto-occipital fasciculus L	Baseline	0.45	0.41	0.53	0.46	0.43
	Follow-up	0.46	0.39	0.46^*^	0.48	0.40
Inferior fronto-occipital fasciculus R	Baseline	0.47	0.42	0.47	0.50	0.42
	Follow-up	0.48	0.41	0.49	0.52	0.45
Inferior longitudinal fasciculus L	Baseline	0.45	0.36	0.49	0.41	0.41
	Follow-up	0.44	0.36	0.47	0.42	0.40
Inferior longitudinal fasciculus R	Baseline	0.47	0.37	0.50	0.41	0.41
	Follow-up	0.44	0.38	0.51	0.43	0.40
Superior longitudinal fasciculus L	Baseline	0.40	0.31	0.42	0.37	0.37
	Follow-up	0.39	0.32	0.42	0.38	0.37
Superior longitudinal fasciculus R	Baseline	0.39	0.32	0.43	0.32	0.36
	Follow-up	0.39	0.32	0.42	0.32	0.35
Uncinate fasciculus L	Baseline	0.46	0.39	0.49	0.40	0.38
	Follow-up	0.42	0.39	0.48	0.37	0.39
Uncinate fasciculus R	Baseline	0.43	0.35	0.42	0.38	0.36
	Follow-up	0.40	0.35	0.43	0.39	0.41^*^

*More than a 10% difference between baseline and follow-up is indicated by an asterisk. L, left; R, right*.

## Discussion

The aim of this study was to explore brain changes in phFTD over a 3-years time period using advanced neuroimaging techniques, and to report these brain changes as well as cognitive changes by means of a descriptive case series. It is of note that due to the qualitative nature of the study, strong conclusions cannot be drawn, but the results however can and should be used as a motivation for further studies. We chose to highlight any individual changes over 10%, to separate these from cases with only minimal changes and reduce the risk of overinterpretation. Clinically, no clear progression was observed after 3 years in phFTD cases with disease durations ranging from 8 to 20 years. Neuropsychological examination showed—similar to baseline—that cognitive profiles may be normal or may be suggestive of bvFTD, and that over time they may remain stable, or show some progression of existing cognitive abnormalities, yet still not fulfilling criteria for a full bvFTD profile. Conventional MRI showed no atrophy, or only unspecific atrophy that did not or only mildly progress. Qualitative comparison of cognitive performance at baseline and follow-up showed some progression in the language and memory domains, yet all cognitive domain scores were still in between those of bvFTD and controls. Qualitative comparison of advanced MRI measures showed that structural brain abnormalities in phFTD showed no evident progression and were generally in between bvFTD and controls, and that functional brain abnormalities, i.e., hyperperfusion, progressed over time and were more pronounced than in bvFTD and controls.

This case series study illustrates that phFTD does not show clear longitudinal clinical changes. However, cognitive domain scores indicated decreased scores for language and memory; this may suggest that progression of these domains clinically may become apparent before that of other cognitive domains, although their scores were still in between those of bvFTD and controls. Additionally, behavioral changes were reported subjectively by the partner and/or patient, but these were not evidently supported by neuropsychological testing, making their interpretation difficult. Importantly, this study also suggests that structural abnormalities, such as GM volume and WM microstructure, appear both to be relatively stable with measures generally in between bvFTD and controls, whereas functional abnormalities do indicate longitudinal changes. Higher perfusion was observed for the whole brain and for each lobe, which is in line with our previous findings (15). A firm conclusion cannot be drawn, as we could not perform any formal comparisons with controls and baseline measurements due to the small sample. However, we can speculate on this finding, as previous literature has hypothesized that functional changes may be related to a mechanism compensating for subtle neuronal dysfunctioning (41, 42). In order for the brain to maintain performance at its normal level, despite such subtle neuronal dysfunctioning, a higher level of perfusion is required. As the neuronal dysfunctioning increases, this mechanism will ultimately fail, resulting in both hypoperfusion as well as diminished performance noticeable at a clinical level. Our hypothesis here is that there is very mild deterioration of neuronal functioning over time in phFTD, which is not clinically obvious due to such a compensatory mechanism at this time.

This case series study also points out the diversity of the phFTD clinical profile and the difficulties with diagnosis. For instance, a long disease duration, behavioral profiles, cognitive profiles and conventional MRI were similar amongst all cases, but were not the same. A specific example is case 2 who, in comparison with other cases, showed lower GM volumes, lower neuropsychological scores, higher perfusion and lower FA. Together with mild clinical progression, we could speculate that this patient is in a slightly more advanced disease stage than the other cases. However, disease duration is 12 years, which is still very much unlike bvFTD. This emphasizes the need for long-term follow-up, with repeated assessments to understand disease development in this particular case, and in phFTD in general.

As bvFTD and phFTD patients were originally included in different research studies, bvFTD patients and part of the control group were scanned on a different scanner. This may have induced slight variation in acquisition, such as in label efficiency in the ASL scans. However, although a scanner effect cannot be excluded entirely, a possible bias is expected to be minimal, as the scanners were of the same type and field strength, and scans were acquired with identical scan protocols. Importantly, the longitudinal measurements in phFTD were all acquired on the same scanner. Additionally, 3-years follow-up data for bvFTD patients and controls were not available for the present study, which may mean that possible changes due to aging have not been accounted for (43). Of note is that the structural measures in phFTD remained stable over time. Also, perfusion has been found to decrease with age rather than increase (44–47), suggesting the observed higher perfusion in the current study is unrelated to an age effect. Additionally with respect to cognition, the observed change in memory is small and it is difficult to be certain this is unrelated to aging, however, given the extent of change in the language domain, it is expected that this is not due to aging alone.

A remarkable finding was an increase of GM volume in case 6 (10.5% overall GM, 11.05% temporal GM, 11.8% parietal GM). Because this was not observed in the other cases, caution with interpretation is still warranted. The literature around GM volume increase is not straightforward, but there are studies showing GM volume increases after, for example, mind-body or cognitive exercises (48, 49). However, we do not know if the patient carried out such exercises; it was not the aim of the study to assess this.

Our study is qualitative in nature due to the small sample size inherent to rare diseases. This limits us in the amount of detail we can apply to the MR analyses performed and prevents us from applying formal testing. We acknowledge that possibly relevant brain changes remain obscured due to reporting MR findings on a whole-brain and lobar level. However, by doing so, we minimize the risk of over interpretation. Future studies with a higher phFTD sample size are encouraged to investigate structural and functional changes at a more detailed region-of-interest level. Despite the lack of formal statistical testing, we feel that our observations still provide important information: phFTD is a rare and still mostly unexplained syndrome, so any additional information is of great value for further understanding and diagnosis. A few studies—to our knowledge—have investigated longitudinal changes in phFTD. In summary, these studies observed a benign disease course over 3-years follow-up in bvFTD patients without—or borderline—conventional MRI abnormalities at presentation (7); did not observe a reported decline of daily living activities over 12 months in patients with bvFTD symptomatology and without conventional MRI abnormalities (6), found no reported progression in clinical records of a subset of bvFTD patients (8); did not observe progression on conventional MRI and neuropsychological testing over a long follow-up (13–21 years) in cases with bvFTD symptomatology and without original clinical progression over 3-years (50); did not observe a clear pattern of FTD pathology post-mortem in two cases with bvFTD symptomatology and without clinical progression (51); and found very slow progression of symptoms over 20 years in one patient with post-mortem confirmed early-stage FTD and a similar very slow progression rate over 15 years in this patient's son (52). Our study adds to the still limited knowledge of longitudinal changes in phFTD.

In conclusion, phFTD patients extensively assessed at 3-years follow-up show symptomatology similar to bvFTD, but with a relatively stable clinical profile. They showed functional brain changes, stable structural brain abnormalities, and some cognitive deficit progression, with cognition and structure generally in between normal and bvFTD. Overall, we suggest that these observations are in line with the notion that phFTD and bvFTD may belong to the same disease spectrum. Despite the fact that a descriptive case series does not allow for strong conclusions, we may use these observations as motivation and a basis for further longitudinal studies in phFTD, specifically exploring the structural vs. functional brain changes. Future longitudinal studies of phFTD are a necessity for improved insight, accurate diagnosis and appropriate treatment in these patients.

## Data Availability Statement

The datasets for this manuscript are not publicly available because the patients included in this study have not given their permission for this. Requests to access the datasets should be directed to corresponding author, marion.smits@erasmusmc.nl.

## Ethics Statement

The studies involving human participants were reviewed and approved by Medical Ethics Committee Erasmus MC–University Medical Centre Rotterdam. The patients/participants provided their written informed consent to participate in this study.

## Author Contributions

MS, RS, and RM contributed to the conception and design of the study. JS contributed to the patient recruitment. RM, RS, and LJ performed the data acquisition. RM, RS, and EB performed the data processing. RM, AL, and MS contributed to the data and results interpretation. RM wrote the manuscript. All authors contributed to manuscript revision, read, and approved the submitted version.

### Conflict of Interest

The authors declare that the research was conducted in the absence of any commercial or financial relationships that could be construed as a potential conflict of interest.
